# Early outcomes after implementation of *treat all* in Rwanda: an interrupted time series study

**DOI:** 10.1002/jia2.25279

**Published:** 2019-04-16

**Authors:** Jonathan Ross, Jean d'Amour Sinayobye, Marcel Yotebieng, Donald R Hoover, Qiuhu Shi, Muhayimpundu Ribakare, Eric Remera, Marcus A Bachhuber, Gad Murenzi, Vincent Sugira, Denis Nash, Kathryn Anastos, for Central Africa IeDEA

**Affiliations:** ^1^ Department of Medicine Montefiore Medical Center/Albert Einstein College of Medicine Bronx NY USA; ^2^ Research Division Rwanda Military Hospital Kigali Rwanda; ^3^ Division of Epidemiology College of Public Health Ohio State University Columbus OH USA; ^4^ Department of Statistics and Biostatistics and Institute for Health Health Care Policy and Aging Research Rutgers the State University of New Jersey Piscataway NJ USA; ^5^ Department of Epidemiology and Community Health New York Medical College Valhalla NY USA; ^6^ Rwanda Biomedical Center Kigali Rwanda; ^7^ Institute for Implementation Science in Population Health City University of New York New York NY USA

**Keywords:** HIV, Treat All, universal test and treat, antiretroviral therapy, retention in care, Africa

## Abstract

**Introduction:**

Nearly all countries in sub‐Saharan Africa have adopted policies to provide antiretroviral therapy (ART) to all persons living with HIV (Treat All), though HIV care outcomes of these programmes are not well‐described. We estimated changes in ART initiation and retention in care following Treat All implementation in Rwanda in July 2016.

**Methods:**

We conducted an interrupted time series analysis of adults enrolling in HIV care at ten Rwandan health centres from July 2014 to September 2017. Using segmented linear regression, we assessed changes in levels and trends of 30‐day ART initiation and six‐month retention in care before and after Treat All implementation. We compared modelled outcomes with counterfactual estimates calculated by extrapolating baseline trends. Modified Poisson regression models identified predictors of outcomes among patients enrolling after Treat All implementation.

**Results:**

Among 2885 patients, 1803 (62.5%) enrolled in care before and 1082 (37.5%) after Treat All implementation. Immediately after Treat All implementation, there was a 31.3 percentage point increase in the predicted probability of 30‐day ART initiation (95% CI 15.5, 47.2), with a subsequent increase of 1.1 percentage points per month (95% CI 0.1, 2.1). At the end of the study period, 30‐day ART initiation was 47.8 percentage points (95% CI 8.1, 87.8) above what would have been expected under the pre‐Treat All trend. For six‐month retention, neither the immediate change nor monthly trend after Treat All were statistically significant. While 30‐day ART initiation and six‐month retention were less likely among patients 15 to 24 versus >24 years, the predicted probability of both outcomes increased significantly for younger patients in each month after Treat All implementation.

**Conclusions:**

Implementation of Treat All in Rwanda was associated with a substantial increase in timely ART initiation without negatively impacting care retention. These early findings support Treat All as a strategy to help achieve global HIV targets.

## 1 Introduction

Initiating antiretroviral therapy (ART) soon after HIV diagnosis substantially decreases HIV‐related morbidity, all‐cause mortality and HIV transmission 1, 2, 3. Accordingly, in 2015 the World Health Organization (WHO) recommended immediate provision of ART to all people living with HIV (PLWH) (“Treat All”) 4. In sub‐Saharan Africa, the epicentre of the global HIV epidemic, nearly all countries have adopted these guidelines 5. However, to date, limited data from this region exist describing HIV care outcomes after national implementation of Treat All.

While recent large, controlled studies examining Treat All have demonstrated high rates of ART initiation and retention 6, 7, it is not yet clear whether these findings will translate into uncontrolled routine settings. Significant questions remain about whether programmes will enrol patients on ART quickly, whether patients will subsequently remain in care, and which factors could affect these outcomes. Under earlier treatment guidelines, individual factors associated with failure to initiate or continue ART have included younger age, male sex and high CD4 count at enrolment 8, 9, 10, 11, 12, 13, 14. Facility‐level factors including geographical location, clinic size and availability of services may also influence ART initiation and retention in care 15, 16. As HIV programmes scale up treatment under Treat All, understanding factors associated with initiating ART and remaining in care will be essential to identify optimal strategies for programme implementation.

On 1 July 2016 Rwanda, a small East African nation with a population of 12 million, became one of the first sub‐Saharan African countries to implement Treat All nationally. Using routinely collected patient‐ and health centre‐level data from ten Rwandan health centres, we aimed to estimate the effect of Treat All implementation on timely ART initiation and retention in care, as well as identify predictors of ART initiation and retention in care after Treat All implementation.

## 2 Methods

### 2.1 Study design

To study the impact of Treat All implementation in July 2016, we conducted an interrupted time series analysis of clinical data from July 2014 through November 2017. We utilized routinely collected data from an open observational cohort of patients receiving HIV care at ten Rwandan health centres affiliated with the Central Africa International epidemiologic Databases to Evaluate AIDS (CA‐IeDEA; www.iedea-ca.org). CA‐IeDEA is a multi‐country project that collects secondary data from patients receiving HIV care and treatment in the Central African region and is one of seven regions that comprise the global IeDEA network (www.iedea.org). The ten health centres in Rwanda have been previously described 17. All research was conducted according to the principles of the Helsinki Declaration and was approved by the Rwanda National Ethics Committee and the Albert Einstein College of Medicine Institutional Review Board, both of which waived written or verbal patient consent because the data were de‐identified prior to extraction into research database. This study is reported in accordance with the STROBE statement for reporting of observational studies (Table S1).

### 2.2 Population and setting

We included all persons ≥15 years of age newly enrolling in care at health centres affiliated with CA‐IeDEA from 1 July 2014 through 13 September 2017 (90 days prior to the close of dataset). Because we focused on patients newly initiating HIV care, persons known to have transferred from another facility (N=323), as well as participants receiving HIV care >30 days prior to enrolment, and thus likely to be transfers in (N=415), were excluded (Figure S1). In July 2014, Rwanda had fully implemented guidelines recommending provision of ART to all adults (≥15 years) with CD4 count <500 cells/mm^3^, as well as all pregnant or breastfeeding women and all patients co‐infected with tuberculosis or viral hepatitis 18. In July 2016, national HIV treatment guidelines were expanded to include ART for all persons with HIV regardless of disease stage or CD4 count 19; all health centres included in this analysis reported implementation of these guidelines in July 2016. The 2016 guidelines also recommended ART initiation within seven days of diagnosis, attending medical consultations every three months, and monthly ART pick‐up from health centre pharmacies.

### 2.3 Data sources

Each participating health centre routinely collects demographic, clinical and laboratory data as part of clinical care using standardized paper forms; these data are regularly entered into electronic databases. Patient data were de‐identified prior to extraction into the research database. Health centre characteristics were obtained as part of a site assessment periodically conducted at all sites participating in the global IeDEA network 20.

### 2.4 Outcomes and predictor variables

Two primary outcomes were considered in this analysis: ART initiation within 30 days of enrolment and six‐month retention in care. We used the date of enrolment into HIV care as specified in health centre data; we defined ART initiation date as the date of the first ART prescription ordered after enrolment. All patients were included in analyses of ART initiation. We defined six‐month retention as having at least one health centre visit within five to nine months after enrolment. All patients whose enrolment was more than nine months before the close of the dataset and were not known to have died or transferred out prior to the six‐month visit window were included in analyses of this outcome. As secondary outcomes, we also examined the proportion of patients ever initiating ART and the number of days between enrolment and ART initiation. Because viral load measurement was performed on <10% of patients who entered care in the pre‐Treat All period and <50% of those entering care during the Treat All period, we did not analyse viral suppression as an outcome.

Baseline demographic and clinical variables included sex (female or male), age, body mass index (<18.5 vs. ≥18.5 kg/m^2^), referral source into HIV care (voluntary counselling/testing programme (VCT), maternal/prenatal care, other), WHO stage (I‐II vs. III‐IV) and CD4 count (categorized as <200, 200 to 349, 350 to 500 and ≥500 cells/μL), measured up to 90 days after enrolment. Health centre characteristics included location (urban vs. peri‐urban), size (≥2000 vs. <2000 patients with HIV in care), whether adolescents and adults were seen in separate clinics or patients of all ages were seen together, availability of physicians, mid‐level providers and adherence counsellors (all or some of the time vs. none of the time), whether sites provided incentives (such as mobile phone airtime vouchers or transportation costs) for early enrolment in care, the number of pre‐ART counselling sessions typically occurring at the health centre (four or more vs. less than four), and availability of adherence support including medication review and referrals to mental health counselling or peer support. For all variables, missing values were categorized as such.

### 2.5 Analyses

We defined two periods for the study: the pre‐Treat All period (July 2014 through June 2016), and the Treat All period (July 2016 through November 2017). Baseline characteristics of patients enrolling in the pre‐Treat All and Treat All periods were compared using bivariate logistic regression models that accounted for clustering within health centres. We used the Kaplan–Meier method to estimate median time from enrolment to ART initiation.

For the interrupted time series analysis, we used segmented linear regression models to estimate the predicted probability of initiating ART within 30 days of enrolment and six‐month retention in care among patients entering care in each month. To do this, we fit a separate model for each outcome as follows:

$$\begin{array}{cc} Y_{t}=\; & \beta_{0}+\beta_{1}\times pre-TreatAlltrend+\beta_{2}\times \mathrm{TreatAllchange}\\ & +\beta_{3}\times \mathrm{TreatAlltrend}+\varepsilon_{t} \end{array}$$

In these models, Yt is the independent outcome (predicted probability), *β*_0_ estimates the baseline level at the beginning of the study period, *β*_1_ *estimates* the linear trend before Treat All implementation, *β*_2_ estimates the immediate level change (i.e. jump) after Treat All implementation and *β*_3_ estimates the change in linear trend after Treat All implementation relative to the pre‐Treat All trend. Models accounted for clustering within health centres and did not include data on patients enrolling in care from 1 June to 31 July 2016 to account for a two‐month transition period of Treat All guideline implementation.

For both primary outcomes, we plotted the proportion of patients enrolling in each month who achieved each outcome as well as fitted values from the segmented regression models described above. We also calculated counterfactual values by extending the pre‐Treat All regression models (i.e. not including the *Treat All change* and *Treat All trend* terms). We then calculated differences between the observed and expected (counterfactual) outcomes at the last month of follow‐up. We calculated 95% confidence intervals (CIs) using the bootstrap method 21. Sub‐analyses were performed using similar models to examine levels and trends by sex, age, referral source and baseline CD4 count.

Finally, we examined predictors of initiating ART within 30 days and of six‐month retention in care among patients enrolling after Treat All guidelines. For these analyses we utilized modified Poisson regression models with robust variances to calculate crude and adjusted risk ratios (RRs), with generalized estimating equations to account for clustering within health centres. Multivariate models were adjusted for all individual demographic and clinical characteristics but were not adjusted for health centre characteristics given the relatively small number of centres (N=10). Data were analysed using SAS 9.4 (SAS Institute Inc., Cary, NC); statistical significance for all tests was two‐sided at *p* < 0.05.

In sensitivity analyses, to account for differences in ART eligibility criteria, we examined the proportion of patients initiating ART and time from enrolment to ART initiation excluding patients enrolling in care before Treat All who were not eligible for ART (i.e. those with CD4 500 cells/mm^3^). We then repeated the segmented regression analysis of ART initiation within 30 days limited to patients eligible for ART. Similarly, to account for the potential influence of ART initiation on retention in care, we repeated the segmented regression analysis of six‐month retention in care limited to patients who initiated ART. Finally, to determine whether use of missing indicators biased results, we modelled predictors of ART initiation within 30 days and six‐month retention in care using a complete case analysis that excluded missing values.

### 2.6 Role of the funding source

The funders of the study had no role in the study design, data collection, data analysis, data interpretation or writing of the report. The corresponding author had full access to all the data in the study and had final responsibility for the decision to submit for publication.

## 3 Results

In total, 2885 patients were included in this analysis, of whom 1803 (62.5%) entered care during the pre‐Treat All period and 1082 (37.5%) during the Treat All period (Table 1). Most (59.1%) were female and median age was 32 years in both groups. Patients enrolling during the Treat All period were more likely to be missing CD4 count at baseline (*p* =0.05); otherwise demographic and clinical characteristics did not differ statistically between the two groups. Among the ten health centres examined, seven were located in urban or peri‐urban areas, four had >2000 patients in HIV care and four had separate HIV clinics for adolescents and adults (Table S2). All health centres had mid‐level clinicians and adherence counsellors; half were staffed at least some of the time by a physician.

**Table 1:** Baseline characteristics of 2885 patients enrolling in care in ten health centres in Rwanda, 2014 to 2017

	Enrolled during pre‐Treat All period (July 2014 to June 2016) (N=1803)	Enrolled during Treat All period (July 2016 to September 2017) (N=1082)	*p* value
Sex, n (%)			
Female	1090 (60.5)	615 (56.8)	0.32
Male	713 (39.5)	467 (43.2)	
Age group, n (%)			
15 to 24 years	297 (16.5)	154 (14.2)	0.09
>24 years	1506 (83.5)	928 (85.8)	
Median age in years (IQR)	32 (26 to 39)	33 (27 to 39)	0.57
Referral source, n (%)			
Voluntary counselling and testing (VCT)	1266 (70.2)	714 (66.0)	0.59
Maternal/prenatal health	276 (15.3)	138 (12.8)	
Other^a^	202 (11.2)	115 (10.6)	
Missing	59 (3.3)	115 (10.6)	
BMI, n (%)			
<18.5 kg/m^2^	310 (17.2)	149 (13.8)	0.25
≥18.5 kg/m2	1368 (75.9)	839 (77.5)	
Missing	125 (6.9)	94 (8.7)	
WHO HIV stage, n (%)			
Stage I‐II	1565 (86.8)	914 (84.5)	0.10
Stage III‐IV	160 (8.9)	90 (8.3)	
Missing	78 (4.3)	78 (7.2)	
CD4 cell count, n (%)			
≥500 cells/μL	598 (33.2)	287 (26.5)	0.05
350 to 499 cells/μL	347 (19.2)	181 (16.7)	
200 to 349 cells/μL	319 (17.7)	180 (16.6)	
<200 cells/μL	325 (18.0)	180 (16.6)	
Missing	214 (11.9)	254 (23.5)	
Median CD4 count in cells/μL (IQR)	415 (235 to 615)	392 (219 to 586)	0.26
Died in first six months after enrolment, n (%)	27 (1.5)	11 (1.0)	0.27
Transferred out in first six months after enrolment, n (%)	111 (6.2)	50 (4.6)	0.14

BMI, body mass index; IQR, interquartile range; VCT, voluntary counselling and testing; WHO, World Health Organization.

a Other includes tuberculosis programme, referral from primary care, referral from inpatient hospitalization, sex worker outreach, mobile VCT.

Among 1803 patients entering HIV care during the pre‐Treat All period, 1579 (87.6%) initiated ART during the study period, compared to 1032 of 1082 (95.4%) patients entering care during the Treat All period (*p* <0.0001). Median time from enrolment to ART initiation was 31 days among patients in the pre‐Treat All period (interquartile range (IQR) 7 to 158) versus 7 days (IQR 1 to 15) among patients entering care during the Treat All period. When limiting analyses to patients eligible for ART, 963 of 1029 (93.6%) patients entering care in the pre‐Treat All period initiated ART, compared to 1032 of 1082 (95.4%) entering care in the Treat All period (*p* =0.07). In this restricted analysis, median time to ART was 23 days (IQR 7 to 48) in the pre‐Treat All period versus 6 days (IQR 0 to 14) during the Treat All period (*p* <0.0001).

**Figure 1:**
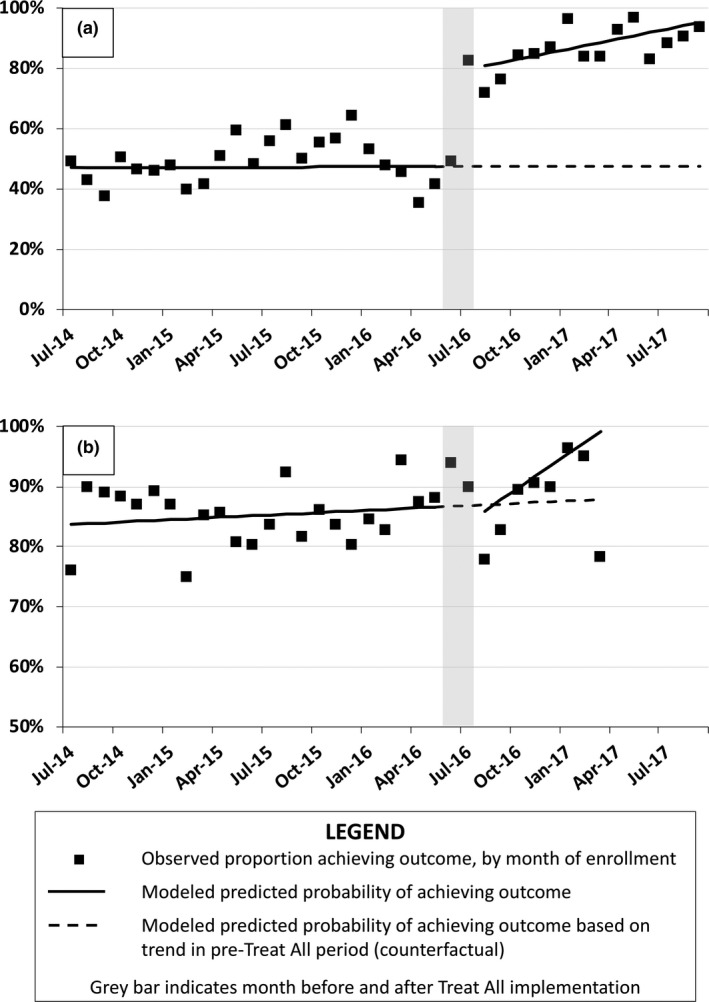
Proportion of patients (a) initiating antiretroviral therapy within 30 days of enrolment and (b) retained in care 6 months after enrolment in 10 health centres in Rwanda, 2014 to 2017.

Among patients who entered care in July 2014 (the beginning of the pre‐Treat All period), the predicted probability of initiating ART within 30 days was 47.1% (95% CI 38.2, 56.1); this did not change during the pre‐Treat All period (Figure 1; Table 2). Immediately after Treat All implementation, the predicted probability of initiating ART within 30 days increased to 78.4% (absolute change of 31.3 percentage points, 95% CI 15.5, 47.2), and continued to increase by 1.1 percentage points in each subsequent month (95% CI 0.1, 2.1). At the end of the study period, the predicted probability was 95.2%, or 47.8 percentage points (95% CI 8.1, 87.8) higher than would have been expected under the pre‐Treat All trend.

**Table 2:** Level and trend changes in predicted probabilities^a^ of ART initiation within 30 days of enrolment and six‐month retention in care before and after implementation of Treat All in 10 health centres in Rwanda

	Pre‐Treat All period (July 2014 to May 2016)		Treat All period (August 2016 to September 2017)	
	Baseline^b^ (%) (95% CI)	Pre‐Treat All Trend^c^ (Δ%) (95% CI)	Treat All Change^d^ (%) (95% CI)	Treat All Trend^e^ (Δ%) (95% CI)
ART initiation within 30 days				
Overall	47.1 (38.2, 56.1)	0.0 (−0.6, 0.6)	31.3 (15.5, 47.2)	1.1 (0.1, 2.1)
Sex				
Men	41.1 (31.6, 50.5)	0.1 (−0.7, 1.0)	35.0 (13.6, 56.4)	0.7 (−0.5, 1.9)
Women	51.0 (40.8, 61.2)	−0.1 (−0.7, 0.5)	28.5 (14.4, 42.5)	1.4 (0.2, 2.5)
Age group				
15 to 24 years	47.5 (35.5, 59.4)	0.1 (−0.1, 1.0)	24.0 (2.0, 46.0)	1.4 (0.0, 2.8)
>24 years	46.8 (37.8, 55.7)	0.0 (−0.6, 0.6)	33.5 (18.5, 48.5)	0.9 (−0.4, 2.3)
Referral source				
VCT	38.8 (27.9, 49.7)	0.0 (−0.5, 0.8)	32.9 (13.8, 51.9)	1.2 (−0.1, 2.6)
Maternal/prenatal	69.3 (62.2, 76.5)	0.5 (−0.1, 1.1)	6.0 (−14.5, 26.5)	0.0 (−0.7, 0.7)
Other	49.9 (39.0, 60.7)	0.4 (−0.4, 1.2)	29.4 (10.5, 48.3)	−0.7 (−2.2, 0.8)
Baseline CD4 count				
>500 cells/μL	35.9 (28.6, 43.1)	−0.2 (−0.8, 0.4)	52.2 (36.2, 68.3)	1.1 (−0.1, 2.3)
350 to 500 cells/μL	50.7 (35.5, 65.8)	0.5 (0.0, 0.9)	19.4 (5.1, 33.7)	1.0 (−0.3, 2.3)
200 to 349 cells/μL	63.3 (46.7, 80.0)	−0.5 (−1.6, 0.6)	26.7 (−0.3, 53.6)	2.4 (0.3, 4.5)
<200 cells/μL	53.6 (37.1, 70.1)	0.5 (−0.7, 1.7)	19.2 (−4.7, 43.1)	−0.8 (−2.8, 1.2)
Missing	35.3 (28.2, 42.4)	0.0 (−0.1, 1.0)	32.4 (13.0, 51.9)	1.2 (−0.6, 3.0)
Six‐month retention in care				
Overall	83.7 (78.7, 88.8)	0.1 (−0.1, 0.4)	−4.5 (−18.9, 9.9)	1.8 (−1.5, 5.0)
Sex				
Men	87.6 (82.6, 92.6)	−0.3 (−0.6, 0.1)	4.3 (−12.5, 21.1)	1.4 (−2.6, 5.5)
Women	81.4 (75.1, 87.7)	0.4 (0.1, 0.7)	−10.8 (−24.8, 3.2)	2.3 (−0.4, 5.0)
Age group				
15 to 24 years	80.5 (73.6, 87.3)	0.1 (−0.5, 0.7)	−17.3 (−38.3, 3.7)	6.8 (2.7, 10.8)
>24 years	84.3 (79.2, 89.3)	0.1 (−0.1, 0.4)	−2.5 (−16.7, 11.7)	1.0 (−2.3, 4.3)
Referral source				
VCT	87.1 (81.8, 92.4)	0.0 (−0.4, 0.3)	−4.4 (−22.5, 13.7)	2.1 (−1.8, 5.9)
Maternal/prenatal	81.8 (74.5, 89.1)	0.2 (−0.2, 0.7)	−6.5 (−31.2, 18.3)	1.0 (−4.4, 6.5)
Other	72.8 (65.9, 79.7)	0.8 (0.5, 1.2)	−16.3 (−34.0, 1.2)	2.9 (−0.1, 6.0)
Baseline CD4 count				
>500 cells/μL	85.7 (79.4, 91.9)	−0.1 (−0.5, 0.4)	0.4 (−14.6, 15.4)	1.5 (−1.5, 4.5)
350 to 500 cells/μL	89.5 (79.5, 99.4)	−0.2 (−0.8, 0.4)	−13.2 (−39.4, 13.0)	4.7 (−1.2, 10.7)
200 to 349 cells/μL	92.3 (87.5, 97.1)	0.1 (−0.3, 0.4)	5.1 (1.3, 8.8)	−0.2 (−0.7, 0.2)
<200 cells/μL	84.3 (73.5, 95.1)	0.1 (−0.6, 0.9)	−7.2 (−21.6, 7.2)	3.3 (1.0, 5.7)
Missing	54.2 (38.3, 70.0)	1.1 (−0.1, 2.2)	0.4 (−18.9, 19.6)	−0.8 (−9.0, 7.5)

ART, antiretroviral therapy; VCT, voluntary counselling and testing.

^a^Probabilities modelled using segmented linear regression models: *predicted probability = Baseline + β*_1_ *×Pre‐Treat All Trend + β*_2_ *×Treat All Change + β*_3_ *×Treat All Trend*; ^b^refers to the predicted probability of outcome at the beginning of the study period, *β*_0_ *;* ^c^refers to the modelled change in predicted probability of outcome per month during the pre‐Treat All period, *β*_1_; ^d^refers to the modelled change in predicted probability of outcome immediately after implementation of Treat All compared to immediately before implementation, *β*_2_; ^e^Refers to the modelled difference in trend in predicted probability relative to the pre‐Treat All period, *β*_3_.

The predicted probability of six‐month retention in care among patients who entered care in July 2014 was 83.7% (95% CI 78.7, 88.8); this increased non‐significantly by 0.1 percentage points (95% CI −0.1, 0.4) during each month of the pre‐Treat All period. Immediately after Treat All implementation, the predicted probability of six‐month retention in care changed by −4.5 percentage points (95% CI −19.9, 9.9), and this increased non‐significantly by 1.8 percentage points (95% CI −1.5, 5.0) during each month after Treat All implementation. When we compared observed and expected estimates from the regression model at the end of the study period, the predicted probability of six‐month retention in care was 94.9%, or 11.2 percentage points higher (95% CI −23.8, 46.2) than would have been expected under the pre‐Treat All trend.

For the above analyses, similar estimates for immediate changes after Treat All implementation, as well as trends before and after Treat All implementation were observed in sensitivity analyses limited to patients eligible for ART (for ART initiation within 30 days) and patients on ART (for 6‐month retention in care; Table S3).

Of 1082 patients enrolling in care during the Treat All period, ART initiation within 30 days was slightly more likely among patients referred from maternal/prenatal health or other settings (aRR 1.07, 95% CI 1.02, 1.13) compared to VCT, was less likely among patients aged 15 to 24 compared to >24 years (aRR 0.93, 95% CI 0.87, 1.00) (Table 3). Among 709 patients with ≥9 months between enrolment and the close of the dataset, six‐month retention in care was higher among patients initiating ART within 30 days compared to not initiating ART within 30 days or not at all (aRR 1.15, 95% CI 1.07, 1.24) and in health centres providing incentives for early enrolment in care compared to those that did not (aRR 1.08, 95% CI 1.01, 1.15), whereas retention was lower among patients aged 15 to 24 years compared to those >24 years (aRR 0.89, 95% CI 0.82, 0.98). In a complete case sensitivity analysis, similar results were observed for both 30‐day ART initiation and six‐month retention in care (Table S4).

**Table 3:** Predictors of ART initiation within 30 days and six‐month retention in care among patients enrolling in care in 10 Rwandan health centres during the Treat All period

	ART initiation within 30 days (N=1082)		Six‐month retention in care (N=559)	
	RR (95% CI)	aRR (95% CI)	RR (95% CI)	aRR (95% CI)
Patient characteristics				
Female (vs. male)	1.00 (0.96, 1.04)	1.00 (0.96, 1.03)	1.00 (0.97, 1.04)	1.03 (0.99, 1.07)
Aged 15 to 24 years (vs. >24 years)	0.94 (0.88, 0.99)*	0.93 (0.87, 1.00)*	0.89 (0.82, 0.97)*	0.89 (0.82, 0.98)*
Referral source, n (%)				
Maternal/prenatal health versus VCT	1.07 (1.02, 1.14)**	1.07 (1.02, 1.13)*	1.01 (0.95, 1.08)	1.00 (0.94, 1.07)
Other^a^ versus VCT	1.03 (0.99, 1.07)	1.04 (1.01, 1.08)*	1.00 (0.86, 1.17)	1.01 (0.88, 1.16)
BMI <18.5 kg/m2 (vs. ≥18.5), n (%)	0.96 (0.89, 1.04)	0.97 (0.90, 1.05)	1.00 (0.92, 1.09)	1.01 (0.93, 1.09)
WHO HIV stage				
Stage III‐IV versus Stage I‐II	0.95 (0.88, 1.02)	0.97 (0.88, 1.07)	1.00 (0.94, 1.07)	0.99 (0.91, 1.07)
Missing versus Stage I	0.88 (0.73, 1.06)	0.95 (0.83, 1.09)	0.89 (0.75, 1.05)	0.87 (0.74, 1.02)
CD4 cell count				
<200 versus ≥500 cells/mm^3^	0.99 (0.92, 1.06)	0.99 (0.91, 1.07)	1.01 (0.97, 1.06)	1.01 (0.97, 1.06)
200 to 349 versus ≥500 cells/mm^3^	1.04 (0.97, 1.11)	1.04 (0.97, 1.11)	0.96 (0.79, 1.15)	0.96 (0.81, 1.13)
350 to 499 versus ≥500 cells/mm^3^	1.03 (0.97, 1.09)	1.03 (0.97, 1.10)	1.09 (1.01, 1.16)*	1.08 (1.01, 1.15)*
Missing versus ≥500 cells/mm^3^	0.92 (0.82, 1.04)	0.94 (0.83, 1.05)	0.90 (0.80, 1.02)	0.92 (0.83, 1.01)
ART initiation <30 days (vs. not initiated in <30 days)	‐	‐	1.18 (1.07, 1.29)**	1.15 (1.07, 1.24)**
Health centre characteristics				
Peri‐urban versus urban	0.88 (0.75, 1.03)	0.91 (0.78, 1.05)	0.91 (0.83, 1.02)	0.94 (0.85, 1.05)
≥2000 HIV patients in care (vs. <2000)	1.08 (0.97, 1.21)	1.07 (0.97, 1.19)	1.00 (0.89, 1.12)	0.99 (0.89, 1.10)
Age‐differentiated clinic (vs. all‐ages clinic)	1.04 (0.91, 1.18)	1.05 (0.93, 1.18)	0.93 (0.84, 1.04)	0.93 (0.84, 1.03)
Physician available some/all of the time (vs. not)	0.97 (0.86, 1.10)	0.98 (0.86, 1.11)	1.06 (0.95, 1.17)	1.06 (0.95, 1.18)
Incentives for early enrolment in care (vs. not)	0.95 (0.84, 1.08)	0.96 (0.86, 1.09)	1.08 (0.98, 1.19)	1.08 (1.01, 1.15)*
4 pre‐ART counselling sessions (vs. < 4)	0.94 (0.83, 1.07)	0.95 (0.84, 1.07)	1.04 (0.93, 1.16)	1.04 (0.94, 1.15)
Types of ART adherence support routinely available (vs. not available)				
Referral to mental health counselling	‐	‐	1.00 (0.90, 1.12)	0.98 (0.91, 1.06)
Referral to peer support	‐	‐	0.96 (0.86, 1.06)	0.94 (0.86, 1.03)
Routine review of medication pickup	‐	‐	0.97 (0.87, 1.08)	0.96 (0.88, 1.05)

ART, antiretroviral therapy; RR, rate ratio; CI, confidence interval; VCT, voluntary counselling and testing; BMI, body mass index; WHO, World Health Organization.

a Other includes tuberculosis programme, referral from primary care, referral from inpatient hospitalization, sex worker outreach, mobile VCT.

* *p* < 0.05; **p < 0.01.

## 4 Discussion

In this analysis of patients enrolling in care at ten health centres in Rwanda, we found that guidelines and policy to implement ART for all people living with HIV infection, known as Treat All, led to a substantial decline in median time from enrolment to ART initiation, a large increase in the proportion of patients initiating ART within 30 days of entering care and a statistically non‐significant increase in the proportion of patients retained in care at six months. These results lend support to Treat All as a viable strategy to expand ART without negatively impacting retention.

We found that Treat All implementation was associated with an immediate 31.3 percentage point increase in the proportion of patients initiating ART within 30 days of enrolment. The proportion of patients initiating ART within 30 days increased significantly with each month after Treat All implementation, with 95.2% of patients at the end the study period achieving this outcome compared to a predicted 47.5% based on linear extension of the pre‐Treat All 30‐day ART initiation trend. Similar results were observed even when excluding patients who were not eligible for ART prior to Treat All implementation, indicating that the change was not solely due to the new guideline and its implementation at the sites, but rather in improved programme capacity to rapidly initiate ART. These data are consistent with successful implementation of earlier ART guidelines in Rwanda leading to rapid increases in median CD4 count at ART initiation, as well as global data showing rapid increases in ART uptake as treatment thresholds increased 17, 22, 23.

We observed a small, non‐significant increase in six‐month retention after Treat All implementation. However, among patients enrolling in care in the Treat All period, retention in care among patients initiating ART within 30 days was significantly higher than those not initiating ART within 30 days after enrolment. These results are consistent with earlier studies that have described either no effect or improvement in retention in care associated with early ART initiation, including preliminary findings from the MaxART trial in eSwatini and from implementation of Treat All in Malawi 24, 25, 26, 27, 28. In sub‐group analyses, retention in care did not significantly worsen after Treat All implementation among those with more advanced HIV (as indicated by CD4 count) or among men and younger patients, who have traditionally been at risk of worse outcomes. Among patients referred to HIV care from maternal/prenatal clinics, there was no significant change in six‐month retention after Treat All implementation, and overall retention at six months was higher than in studies of pregnant women initiating HIV care under Option B+ programmes in other settings 29. Additional research is needed to determine whether the observed results reflect a population‐wide benefit of Treat All, even to at‐risk groups, or may be a function of Rwanda's already high‐performing HIV programme. Nonetheless, taken together, our findings provide early, real‐world evidence that initiating all patients on ART is feasible, and that expanding ART to all patients living with HIV appears to improve treatment uptake without worsening retention in care.

Among patients enrolling in care after Treat All implementation, we did not find an association between baseline CD4 count and 30‐day ART initiation. Prior studies, including the Treatment as Prevention trial, have demonstrated that treatment expansion does not result in delayed ART initiation among sicker patients 22, 30, 31. To our knowledge, our study is the first to confirm these findings within a routine implementation of a Treat All paradigm. Unexpectedly, we found a significant increase in the proportion of patients missing enrolment CD4 counts after Treat All implementation, potentially affecting our estimates. The high frequency of missing data may be a function of less frequent CD4 monitoring as this is no longer needed to determine treatment eligibility, despite the continued recommendation of baseline CD4 measurement in national guidelines 19. Similar findings were observed in South Africa under changes in CD4 monitoring policy 32 and may be expected in other settings as Treat All implementation continues 33, potentially limiting opportunities to evaluate the impact of this policy.

Among patients enrolling in care after Treat All, we also observed negligible differences among patients grouped by baseline CD4 count with respect to six‐month retention in care. This result is in contrast to results from the SEARCH trial, which reported lower rates of retention among patients with CD4 count below country treatment initiation threshold 7. Our findings may be a reflection of consistently high levels of retention in care in Rwanda's HIV programme, even among patients not on ART prior to Treat All 12. This suggests that at the time of Treat All implementation, the programme was already quite adept at retaining patients at various stages of disease. The differences between this study and the SEARCH trial may also be explained by differences in setting and clinical procedures that may affect retention, as the SEARCH trial was conducted in a largely rural setting and included home‐based HIV testing. It is not clear that similar retention outcomes will be observed under Treat All programmes in other routine settings in sub‐Saharan African countries, given the substantial heterogeneity of retention in care in different settings 34, As our study included a relatively smaller number of patients with a short duration of follow‐up (up to 9 months), additional research is necessary to better understand longer term retention under Treat All in Rwanda and elsewhere.

Among patients enrolling in care after Treat All implementation, those aged 15 to 24 were slightly less likely than those >24 years to initiate ART and were less likely to be retained in care at six months. These results are consistent with those from multiple studies conducted under earlier treatment guidelines 35, 36, as well as those from the SEARCH and HPTN 071 trials 7, 37. Despite the worse outcomes observed among patients aged 15 to 24 compared to those >24 years, there was a large increase in the proportion of young patients initiating ART within 30 days during the Treat All period compared to the pre‐Treat All period, with no significant negative impact on retention in care at six months. While additional efforts will be needed to engage and keep adolescents and young adults in care under Treat All, the observed improvements in timely ART initiation are encouraging.

This study has several limitations. First, we only assessed patients at care enrolment rather than at initial HIV diagnosis and were therefore unable to directly examine the impact of Treat All guidelines with respect to WHO guidelines recommending ART as soon as HIV diagnosis is confirmed. As we relied on routinely collected data, we did not have complete information on pregnancy status at enrolment, and thus utilized a proxy measurement of referral from maternal/prenatal health centres. Similarly, the use of routine clinical data limited our ability to examine other factors (e.g. education, substance use) that may influence clinical outcomes. Because the analysis was restricted to patients in care in health centres affiliated with CA‐IeDEA, some patients not retained in care at six months may have died or transferred silently to other health centres, and we were not able to ascertain vital status among those lost to follow‐up. Additionally, estimate precision may have been limited by the relatively small number of patients enrolling in each month as well as the relatively short time since Treat All implementation in Rwanda and the comparatively low number of newly diagnosed patients initiating care. Finally, the cohort consisted of patients enrolling in care at health centres located in or near the capital of a country with a highly functional HIV care service delivery system and with a lower HIV prevalence than in much of southern Africa. This may limit the generalizability of our findings.

## 5 Conclusions

In conclusion, in this study of ten health centres in Rwanda, implementation of Treat All led to substantial improvements in timely ART initiation without negatively impacting retention in care. These are important early data from “real world” Treat All implementation in sub‐Saharan Africa that lend support to this approach as a strategy to help achieve global HIV targets.

## Competing interests

All authors have no conflicts of interest to disclose.

## Authors’ contributions

JR, MY, DH, QS and KA contributed to the study design, data analysis and interpretation. MB contributed to data analysis and interpretation. JS and DN contributed to the study design and interpretation. MR, ER, GM and VS participated in data collection and interpretation. JR drafted the first version of the manuscript, which all authors subsequently reviewed, edited and approved.

## Supporting information


**Table S1.** STROBE (Strengthening The Reporting of OBservational Studies in Epidemiology) Checklist
**Table S2.** Characteristics of 10 health centres in Rwanda included in Analysis
**Table S3.** Level and trend changes in predicted probabilities† of ART initiation within 30 days of enrolment and six‐month retention in care before and after implementation of Treat All in 10 health centres in Rwanda
**Table S4.** Predictors of ART initiation within thirty days and six‐month retention in care among patients enrolling in care in 10 Rwandan health centres during the Treat All period (complete case analysis)

**Figure S1.** STROBE flowchart of patients in Rwanda IeDEA cohort.
