# Supplementary material for: Early outcomes after implementation of treat all in Rwanda: an interrupted time series study
Source: J Int AIDS Soc. 2019 Apr 16;22(4):e25279. doi: 10.1002/jia2.25279 (PMC6468264; doi:10.1002/jia2.25279)
Supplement: Supplementary file 1 — Table S1. STROBE (Strengthening The Reporting of OBservational Studies in Epidemiology) Checklist Table S2. Characteristics of 10 health centres in Rwanda included in Analysis Table S3. Level and trend changes in predicted probabilities† of ART initiation within 30 days of enrolment and six‐month retention in care before and after implementation of Treat All in 10 health centres in Rwanda Table S4. Predictors of ART initiation within thirty days and six‐month retention in care among patients enrolling in care in 10 Rwandan health centres during the Treat All period (complete case analysis) [file JIA2-22-e25279-s001.pdf]

STROBE (Strengthening The Reporting of OBservational Studies in Epidemiology) Checklist

A checklist of items that should be included in reports of observational studies. You must report the page number in your manuscript where you consider each of the items listed in this checklist. If you have not included this information, either revise your manuscript accordingly before submitting or note N/A.

Note: An Explanation and Elaboration article discusses each checklist item and gives methodological background and published examples of transparent reporting. The STROBE checklist is best used in conjunction with this article (freely available on the Web sites of PLoS Medicine at http://www.plosmedicine.org/, Annals of Internal Medicine at http://www.annals.org/, and Epidemiology at http://www.epidem.com/). Information on the STROBE Initiative is available at www.strobe-statement.org.

Section and Item	Item No.	Recommendation	Reported on Page No.
**Title and Abstract**	1	(*a*) Indicate the study's design with a commonly used term in the title or the abstract	1
		(*b*) Provide in the abstract an informative and balanced summary of what was done and what was found	2
**Introduction**			
Background/Rationale	2	Explain the scientific background and rationale for the investigation being reported	3
Objectives	3	State specific objectives, including any prespecified hypotheses	3
**Methods**			
Study Design	4	Present key elements of study design early in the paper	3-4
Setting	5	Describe the setting, locations, and relevant dates, including periods of recruitment, exposure, follow-up, and data collection	4
Participants	6	(*a*) *Cohort study*—Give the eligibility criteria, and the sources and methods of selection of participants. Describe methods of follow-up	4
		Case-control study—Give the eligibility criteria, and the sources and methods of case ascertainment and control selection. Give the rationale for the choice of cases and controls	
		Cross-sectional study—Give the eligibility criteria, and the sources and methods of selection of participants	
		(*b*) *Cohort study*—For matched studies, give matching criteria and number of exposed and unexposed	
		Case-control study—For matched studies, give matching criteria and the number of controls per case	
Variables	7	Clearly define all outcomes, exposures, predictors, potential confounders, and effect modifiers. Give diagnostic criteria, if applicable	5

Section and Item	Item No.	Recommendation	Reported on Page No.
Data Sources/ Measurement	8*	For each variable of interest, give sources of data and details of methods of assessment (measurement). Describe comparability of assessment methods if there is more than one group	4-5
Bias	9	Describe any efforts to address potential sources of bias	7
Study Size	10	Explain how the study size was arrived at	4, S Fig 1
Quantitative Variables	11	Explain how quantitative variables were handled in the analyses. If applicable, describe which groupings were chosen and why	5
Statistical Methods	12	(*a*) Describe all statistical methods, including those used to control for confounding	6-7
		(*b*) Describe any methods used to examine subgroups and interactions	6-7
		(*c*) Explain how missing data were addressed	6-7
		(*d*) *Cohort study*—If applicable, explain how loss to follow-up was addressed	5, 7
		Case-control study—If applicable, explain how matching of cases and controls was addressed	
		Cross-sectional study—If applicable, describe analytical methods taking account of sampling strategy	
		(*e*) Describe any sensitivity analyses	7
**Results**			
Participants	13*	(a) Report numbers of individuals at each stage of study—eg numbers potentially eligible, examined for eligibility, confirmed eligible, included in the study, completing follow-up, and analysed	7-9
		(b) Give reasons for non-participation at each stage	5-7
		(c) Consider use of a flow diagram	S Fig 1
Descriptive Data	14*	(a) Give characteristics of study participants (eg demographic, clinical, social) and information on exposures and potential confounders	7
		(b) Indicate number of participants with missing data for each variable of interest	Table 1
		(c) *Cohort study*—Summarise follow-up time (eg, average and total amount)	
Outcome Data	15*	*Cohort study*—Report numbers of outcome events or summary measures over time	5-7
		Case-control study—Report numbers in each exposure category, or summary measures of exposure	
		Cross-sectional study—Report numbers of outcome events or summary measures	

Section and Item	Item No.	Recommendation	Reported on Page No.
Main Results	16	(*a*) Give unadjusted estimates and, if applicable, confounder-adjusted estimates and their precision (eg, 95% confidence interval). Make clear which confounders were adjusted for and why they were included	5-7, Tab 2-3
		(*b*) Report category boundaries when continuous variables were categorized	5
		(*c*) If relevant, consider translating estimates of relative risk into absolute risk for a meaningful time period	n/a
Other Analyses	17	Report other analyses done—eg analyses of subgroups and interactions, and sensitivity analyses	8-9
**Discussion**			
Key Results	18	Summarise key results with reference to study objectives	9-11
Limitations	19	Discuss limitations of the study, taking into account sources of potential bias or imprecision. Discuss both direction and magnitude of any potential bias	10-12
Interpretation	20	Give a cautious overall interpretation of results considering objectives, limitations, multiplicity of analyses, results from similar studies, and other relevant evidence	12
Generalisability	21	Discuss the generalisability (external validity) of the study results	10-12
**Other Information**			
Funding	22	Give the source of funding and the role of the funders for the present study and, if applicable, for the original study on which the present article is based	12-13

*Give information separately for cases and controls in case-control studies and, if applicable, for exposed and unexposed groups in cohort and cross-sectional studies.

Once you have completed this checklist, please save a copy and upload it as part of your submission. DO NOT include this checklist as part of the main manuscript document. It must be uploaded as a separate file.

Supplemental Table 2. Characteristics of 10 health centers in Rwanda included in analysis

	Urban (N=7)	Peri-urban (N=3)
Clinic size		
Large center (≥ 2000 HIV+ patients)	4	0
Small center (< 2000 HIV+ patients)	3	3
HIV clinic format		
Age-differentiated HIV clinics	2	2
All ages seen together	5	1
Clinical and support staff availability		
Physician available some or all of the time	4	1
Mid-level practitioner available some or all of the time	7	3
Adherence counselor available some or all of the time	7	3
Incentives provided for early enrollment into care	3	1
Typical number of pre-ART adherence counseling sessions		
Four	5	1
< Four	2	2
Adherence support typically available for patients		
Adherence counseling	7	3
Mental health counseling	4	1
Referral to peer support	4	1
Routine review of medication pick-up	4	1

Supplemental Table 3. Level and trend changes in predicted probabilities[†] of ART initiation within 30 days of enrollment and six-month retention in care before and after implementation of Treat All in 10 health centers in Rwanda

		**Pre-Treat All period (July 2014 – May 2016)**		**Treat All period (August 2016 – September 2017)**	
		Baseline[‡] (%) (95% CI)	Pre-Treat All Trend[§] (Δ%) (95% CI)	Treat All Change[¶] (%) (95% CI)	Treat All Trend[#] (Δ%) (95% CI)
**ART initiation within 30 days among patients eligible for ART per national guidelines at time of enrollment**	**Overall**	**51.9 (40.6, 63.2)**	**0.1 (-0.5, 0.8)**	**23.3 (5.2, 41.5)**	**0.9 (-0.1, 1.9)**
	Sex				
	Men	49.2 (38.3, 60.1)	0.0 (-0.8, 0.9)	29.2 (7.3, 51.1)	0.8 (-0.4, 2.0)
	Women	54.3 (42.1, 66.5)	0.2 (-0.4, 0.8)	18.3 (1.8, 34.7)	1.0 (-0.1, 2.1)
	Age group				
	15-24 years	49.5 (33.8, 65.2)	0.4 (-0.9, 1.6)	15.5 (-10.8, 41.8)	1.0 (-0.8, 2.7)
	>24 years	52.3 (40.5, 64.1)	0.1 (-0.6, 0.8)	25.6 (8.4, 42.9)	0.8 (-0.5, 2.2)
	Referral source				
	VCT	46.9 (32.3, 61.5)	0.2 (-0.5, 1.0)	25.9 (4.6, 47.2)	1.1 (-0.3, 2.5)
	Maternal/prenatal	69.1 (57.8, 80.4)	0.7 (-0.3, 1.7)	2.9 (-21.2, 27.0)	-0.2 (-1.4, 1.0)
	Other	58.2 (48.4, 68.0)	0.3 (-0.6, 1.2)	23.4 (4.0, 42.8)	-0.6 (-2.3, 1.1)
	Baseline CD4 count				
	>500 cells/μl	32.5 (19.8, 45.3)	0.7 (-0.1, 2.5)	32.0 (-10.8, 74.8)	0.0 (-2.0, 2.0)
	350-500 cells/μl	50.1 (35.5, 65.8)	0.5 (0.0, 0.9)	19.4 (5.1, 33.7)	1.0 (-0.3, 2.3)
	200-349 cells/μl	63.3 (46.7, 80.0)	-0.5 (-1.6, 0.6)	26.7 (-0.3, 53.6)	2.4 (0.4, 4.5)
	< 200 cells/μl	53.6 (37.1, 70.1)	0.5 (-0.7, 1.6)	19.2 (-4.7, 43.1)	-0.8 (-2.8, 1.2)
	Missing	35.3 (28.2, 42.4)	0.0 (-0.1, 1.1)	32.4 (13.0, 51.9)	1.2 (-0.6, 3.1)
**Six-month retention in care among patients who initiated ART**	**Overall**	**88.0 (82.9, 93.0)**	**0.2 (-0.1, 0.5)**	**-6.8 (-21.9, 8.4)**	**1.5 (-1.9, 4.8)**
	Sex				
	Men	91.7 (87.7, 95.7)	-0.2 (-0.6, 1.3)	1.3 (-16.1, 18.6)	1.0 (-3.0, 5.1)
	Women	85.8 (78.4, 93.2)	0.4 (0.0, 0.8)	-12.5 (-26.6, 1.5)	2.0 (-0.9, 5.1)
	Age group				
	15-24 years	87.4 (80.4, 94.4)	0.2 (-0.3, 0.8)	-23.6 (-43.3, -4.0)	5.9 (1.7, 10.1)
	>24 years	88.2 (83.3, 93.2)	0.1 (-0.1, 0.4)	-3.8 (-18.8, 11.1)	-.8 (-2.6, 4.2)
	Referral source				
	VCT	91.2 (85.7, 96.7)	0.0 (-0.4, 0.4)	-8.1 (-26.7, 10.5)	2.2 (-1.5, 5.9)
	Maternal/prenatal	86.3 (79.3, 93.3)	0.2 (-0.2, 0.6)	-0.6 (-14.4, 13.2)	-0.6 (-4.2, 3.0)
	Other	80.4 (75.1, 85.6)	0.6 (0.3, 0.8)	-7.3 (-16.8, 2.3)	1.7 (0.0, 3.5)

Baseline CD4 count				
>500 cells/µl	88.7 (82.8, 94.7)	0.2 (-0.1, 0.5)	-8.5 (-21.1, 4.1)	1.5 (-1.5, 4.4)
350-500 cells/µl	93.0 (84.8, 100)	-0.2 (-0.7, 0.3)	-17.2 (-43.8, 9.4)	4.5 (-1.2, 10.2)
200-349 cells/µl	92.7 (89.0, 96.3)	0.1 (-0.2, 0.4)	4.4 (0.3, 8.5)	-0.1 (-0.5, 0.3)
< 200 cells/µl	89.3 (80.6, 98.1)	-0.1 (-0.8, 0.6)	-6.2 (-20.2, 7.7)	3.6 (1.2, 6.0)
Missing	59.5 (47.9, 71.1)	1.3 (0.4, 2.2)	2.4 (-21.3, 26.1)	-2.9 (-10.9, 5.2)

ART = antiretroviral therapy; VCT = voluntary counseling and testing

† probabilities modeled using segmented linear regression models: *predicted probability = Baseline +* β_1**Pre-Treat All Trend +* β_2 ** Treat All Change +* β_3 ** Treat All Trend*

‡ Refers to the predicted probability of outcome at the beginning of the study period, β_0

§ Refers to the modeled change in predicted probability of outcome per month during the pre-Treat All period, β_1

¶ Refers to the modeled change in predicted probability of outcome immediately after implementation of Treat All compared to immediately before implementation, β_2

# Refers to the modeled difference in trend in predicted probability relative to the pre-Treat All period, β_3

Supplemental Table 4. Predictors of ART initiation within thirty days and six-month retention in care among patients enrolling in care in 10 Rwandan health centers during the Treat All period (complete case analysis)

	ART initiation within 30 days (N=744)		Six-month retention in care (N=393)	
	RR (95% CI)	aRR (95% CI)	RR (95% CI)	aRR (95% CI)
Patient characteristics				
Female (versus male)	1.00 (0.96, 1.04)	0.99 (0.95, 1.04)	1.00 (0.97, 1.04)	1.03 (0.99, 1.07)
Aged 15-24 years (versus >24 years)	0.94 (0.88, 0.99)*	0.95 (0.85, 1.06)	0.89 (0.82, 0.97)*	0.89 (0.84, 0.95)*
Referral source, n (%)				
Maternal/prenatal health versus VCT	1.07 (1.02, 1.14)**	1.09 (1.05, 1.13)*	1.01 (0.95, 1.07)	1.01 (0.95, 1.07)
Other† versus VCT	1.03 (0.99, 1.07)	1.05 (1.01, 110)*	1.00 (0.86, 1.17)	0.99 (0.91, 1.07)
BMI <18.5 kg/m2 (versus ≥18.5), n (%)	0.96 (0.89, 1.04)	0.99 (0.94, 1.05)	1.00 (0.92, 1.09)	1.08 (1.03, 1.11)*
WHO HIV stage				
Stage III-IV versus Stage I-II	0.95 (0.88, 1.02)	0.95 (0.85, 1.07)	0.99 (0.93, 1.06)	0.97 (0.87, 1.09)
Missing versus Stage I	n/a	n/a	n/a	n/a
CD4 cell count				
<200 versus ≥500 cells/mm3	0.99 (0.92, 1.06)	1.01 (0.93, 1.09)	1.02 (0.98, 1.07)	1.03 (0.99, 1.07)
200-349 versus ≥500 cells/mm3	1.04 (0.97, 1.11)	1.05 (0.98, 1.13)	0.96 (0.81, 1.15)	0.98 (0.84, 1.14)
350-499 versus ≥500 cells/mm3	1.03 (0.97, 1.09)	1.06 (1.00, 1.12)	1.09 (1.02, 1.18)*	1.09 (1.01, 1.19)*
Missing versus ≥500 cells/mm3	n/a	n/a	n/a	n/a
ART initiation <30 days (versus not initiated in <30 days)	-		1.18 (1.07, 1.29)**	1.07 (0.98, 1.26)
Health center characteristics				
Peri-urban versus urban	0.88 (0.75, 1.03)	0.90 (0.76, 1.07)	0.91 (0.83, 1.02)	0.94 (0.86, 1.04)
≥2000 HIV patients in care (versus <2000)	1.08 (0.97, 1.21)	1.04 (0.93, 1.17)	1.00 (0.89, 1.12)	0.99 (0.89, 1.11)
Age-differentiated clinic (versus all-ages clinic)	1.04 (0.91, 1.18)	1.03 (0.92, 1.15)	0.93 (0.84, 1.04)	0.88 (0.80, 0.97)*
Physician available some/all of the time (versus not)	0.97 (0.86, 1.10)	0.98 (0.88, 1.11)	1.06 (0.95, 1.17)	1.03 (0.93, 1.15)
Incentives for early enrollment in care (versus not)	0.95 (0.84, 1.08)	0.98 (0.87, 1.12)	1.08 (0.98, 1.19)	1.09 (1.01, 1.17)*
4 pre-ART counseling sessions (versus < 4)	0.94 (0.83, 1.07)	0.96 (0.85, 1.08)	1.07 (0.97, 1.19)	1.07 (0.98, 1.16)
Types of ART adherence support routinely available (versus not available)				
Referral to mental health counseling	-	-	1.00 (0.90, 1.12)	0.98 (0.89, 1.08)
Referral to peer support	-	-	0.96 (0.86, 1.06)	0.91 (0.83, 1.00)
Routine review of medication pickup	-	-	0.97 (0.87, 1.08)	0.98 (0.89, 1.08)

ART = antiretroviral therapy; RR = rate ratio; CI = confidence interval; VCT = voluntary counseling and testing; BMI = body mass index; WHO = World Health Organization

† Other includes tuberculosis program, referral from primary care, referral from inpatient hospitalization, sex worker outreach, mobile VCT

*P <0.05; **P <0.01
