# Supplementary figures and images for: Early outcomes after implementation of treat all in Rwanda: an interrupted time series study
Source: J Int AIDS Soc. 2019 Apr 16;22(4):e25279. doi: 10.1002/jia2.25279 (PMC6468264; doi:10.1002/jia2.25279)

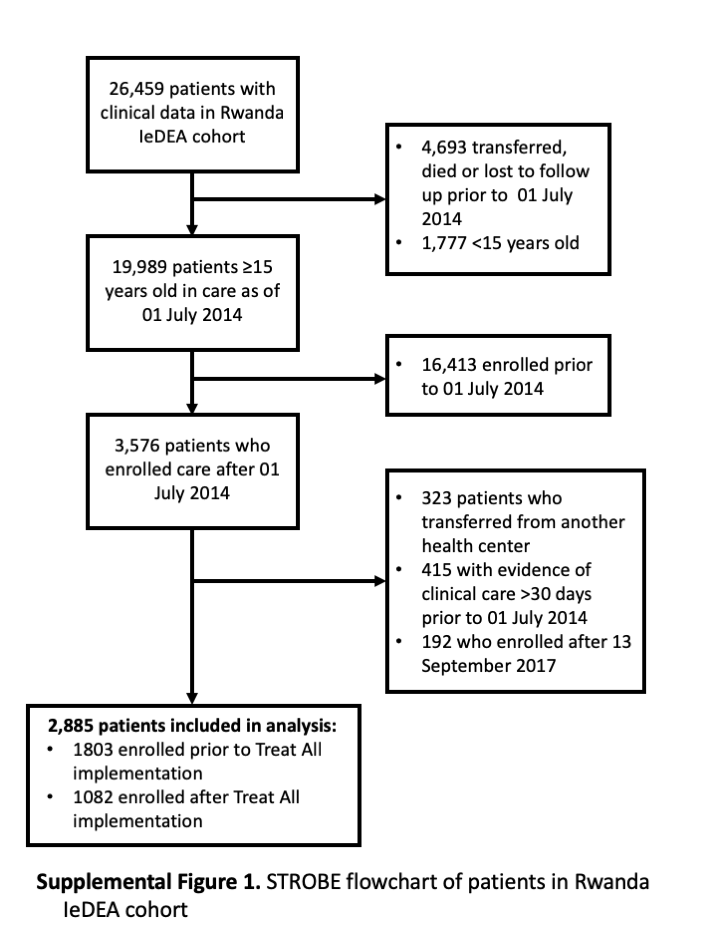

Supplement: Supplementary file 2 — Figure S1. STROBE flowchart of patients in Rwanda IeDEA cohort. [file JIA2-22-e25279-s002.tiff]
